# Up-Regulation of PAI-1 and Down-Regulation of uPA Are Involved in Suppression of Invasiveness and Motility of Hepatocellular Carcinoma Cells by a Natural Compound Berberine

**DOI:** 10.3390/ijms17040577

**Published:** 2016-04-16

**Authors:** Xuanbin Wang, Ning Wang, Hongliang Li, Ming Liu, Fengjun Cao, Xianjun Yu, Jingxuan Zhang, Yan Tan, Longchao Xiang, Yibin Feng

**Affiliations:** 1 Laboratory of Chinese Herbal Pharmacology, Oncology Center, Renmin Hospital, Hubei University of Medicine, 39 Chaoyang Mid-Road, Shiyan 442000, China; wangxb@hbmu.edu.cn (X.W.); hongliangli@hbmu.edu.cn (H.L.); liuming@hbmu.edu.cn (M.L.);fengjuncao@hbmu.edu.cn (F.C.); xjyu@hbmu.edu.cn (X.Y.); zhangjx@hbmu.edu.cn (J.Z.); tanyan@hbmu.edu.cn (Y.T.); longchaoxiang@hbmu.edu.cn (L.X.); 2 Laboratory of Wudang Local Chinese Medicine Research, Hubei University of Medicine, 30 South Renmin Road, Shiyan 442000, China; 3 School of Chinese Medicine, LKS Faculty of Medicine, The University of Hong Kong, 10 Sassoon Road, Pokfulam, Hong Kong, China; ckwang@hku.hk

**Keywords:** berberine, migration, invasion, hepatocellular carcinoma, PAI-1, uPA

## Abstract

Hepatocellular carcinoma (HCC) is the second leading cause of cancer-related death and its prognosis remains poor due to the high risk of tumor recurrence and metastasis. Berberine (BBR) is a natural compound derived from some medicinal plants, and accumulating evidence has shown its potent anti-tumor activity with diverse action on tumor cells, including inducing cancer cell death and blocking cell cycle and migration. Molecular targets of berberine involved in its inhibitory effect on the invasiveness remains not yet clear. In this study, we identified that berberine exhibits a potent inhibition on the invasion and migration of HCC cells. This was accompanied by a dose-dependent down-regulation of expression of Cyclooxygenase-2 (COX-2), nuclear factor kappa B (NF-κB), urokinase-type plasminogen activator (uPA) and matrix metalloproteinase (MMP)-9 in berberine-treated HCC cells. Furthermore, berberine inactivated p38 and Erk1/2 signaling pathway in HCC cells. Primarily, this may be attributed to the up-regulation of plasminogen activator inhibitor-1 (PAI-1), a tumor suppressor that can antagonize uPA receptor and down-regulation of uPA. Blockade of uPA receptor-associated pathways leads to reduced invasiveness and motility of berberine-treated HCC cells. In conclusion, our findings identified for the first time that inactivation of uPA receptor by up-regulation of PAI-1 and down-regulation of uPA is involved in the inhibitory effect of berberine on HCC cell invasion and migration.

## 1. Introduction

There were about 782,500 new liver cancer cases and 745,500 related deaths in the world in 2012. Liver cancer is the second cancer-related mortality for men in the globe and hepatocellular carcinoma (HCC) accounts for the primary tumor in liver cancer [1]. HCC is prevalently diagnosed in the Asian population, especially patients in China, due to the highly genetic risk of hepatic virus infection [2]. A variety of treatments have been developed for HCC patients, however, due to the high potential of reoccurrence and distant metastasis, the prognosis of HCC is far from desirable. Discovery and development of novel treatment to restrict HCC is not only necessary but also emerging. Among all searching strategies, discovering novel compounds from medicinal plants remains to be attractive to scientists in China and all over the world [3].

Berberine (BBR) is a component in some Chinese herbs, especially *Coptidis Rhizoma* (*Coptis chinensis* Franch., *Coptis deltoidea* C. Y. Cheng et Hsiao and *Coptis teeta* Wall.) [4]. Other studies and our previous data showed that BBR may induce HCC cell apoptosis [5,6,7,8,9], autophagic cell death [10,11], and block the cell cycle [12]. Berberine may also enhance the effect of other treatments including vincristine [13], rapamycin [14], evodiamine [15]. Recently, berberine was found to inhibit migration, invasion, metastasis and angiogenesis in HCC [16,17,18]. Regarding the underlying mechanisms of blocking metastasis, we previously found that berberine may suppress Id-1 [19] and inhibit Rho GTPases [18].

Extensive studies have revealed the involvement of inflammation responses in the progression of human cancers. Inflammation has been observed across almost all stages of tumor development, including invasion, angiogenesis, and metastasis [20]. As an inflammatory factor, plasminogen activator inhibitor-1 (PAI-1) plays a pivotal role in regulating migration, invasion and angiogenesis in cancer [21,22].Berberine was reported as a potent inhibitor of inflammation [23,24]. However, it remains a matter of importance as to how it involves in HCC and if PAI-1 is related to the inhibitory effect of berberine on migration and invasion of HCC cells. In this study, Bel-7402 and SMMC-7721 cells were used to investigate the effect of BBR on migration and invasion, and the involvement of PAI-1 underlying as action of mechanism of berberine was observed.

## 2. Results

### 2.1. Inhibitory Effects of Berberine on Hepatocellular Carcinoma (HCC) Cells

**Figure 1:**
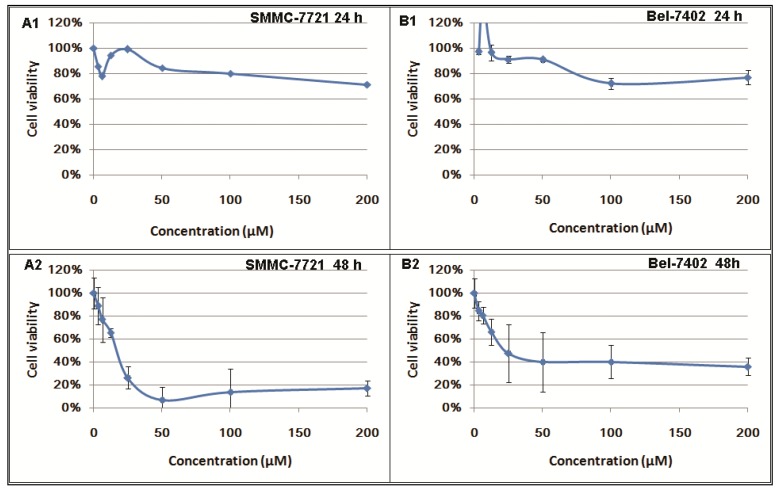

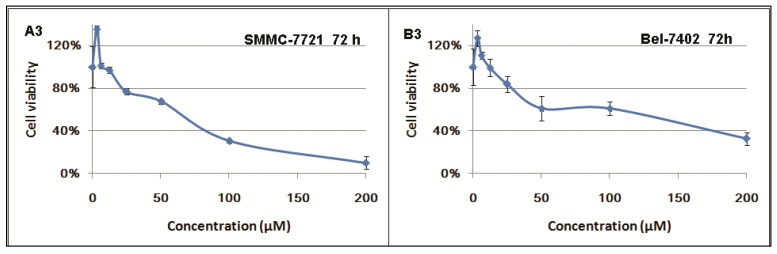
Inhibitory effects of berberine (BBR) on hepatocellular carcinoma (HCC) cells for 24, 48 and 72 h by MTT assay. SMMC-7721 cells were treated with different concentration of BBR (from 0 to 200 μM) for 24 (**A1**), 48 (**A2**) and 72 h (**A3**). Cell viability was detected and calculated; Bel-7402 cells were treated with different concentration of BBR (from 0 to 200 μM) for 24 (**B1**), 48 (**B2**) and 72 h (**B3**). Cell viability was detected and calculated.

Berberine was shown to inhibit tumor cell proliferation and to induce cell death in various kinds of cancer cells [25,26,27,28,29,30,31]. In our study, we investigated the time and dose-manner of berberine on the cell viability of SMMC-7721 and Bel-7402 cells. It was shown that after 24 h treatment, berberine exhibits no significant inhibition on proliferation in HCC cells. Potent cytotoxicity of berberine was observed in cells with 48 and 72 h challenge. At dose higher than 100 μM or when cells were treated more than 24 h, berberine exhibits potent inhibition to both HCC cell lines. (Figure 1).

### 2.2. Intracellular Reactive Oxygen Species (ROS) Production by High Concentration of Berberine in HCC Cells

**Figure 2:**
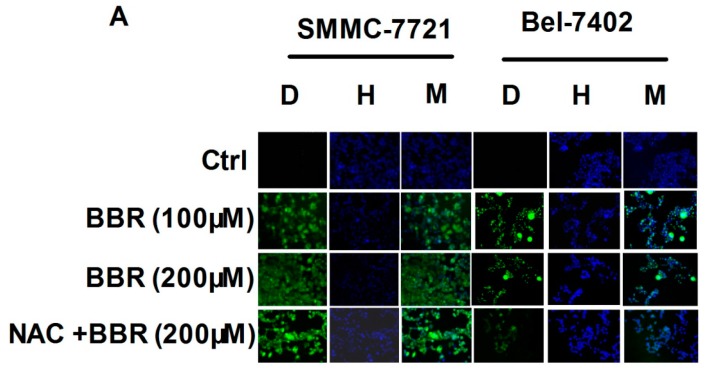

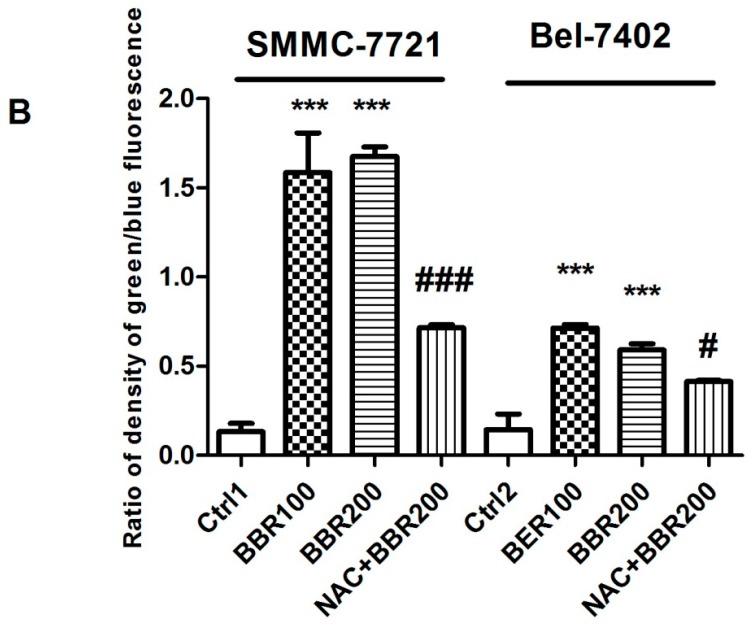
Increased reactive oxygen species (ROS) level in the high concentration of BBR-treated HCC cells by fluorescent microscopy. SMMC-7721 and Bel-7402 cells were incubated in 6-well plates and treated with BBR (100 and 200 μM) for 6 h. 2,7-dichlorodihydrofluorescein diacetate (DCFDA) and Hoechst33258 were used for detecting intracellular ROS level by fluorescent microscopy. Pretreatment of 5 mM of *N*-Acetyl-l-cysteine (NAC) was added as a negative control. (**A**) ROS level was observed by fluorescent microscopy. D: DCFDA (green fluorescence); H: Hoechst33258 (blue fluorescence); M: merge; (**B**) ROS level was calculated by ImageJ 1.38x software (NIH, USA). *** *p* < 0.001 (*vs.* the control group); # *p* < 0.05 (*vs.* BBR200 group); ### *p* < 0.001 (*vs.* BBR200 group).

Intracellular reactive oxygen species (ROS) level was determined as a representative of cellular oxidative stress, which was reported extensively to induce cancer cell death [32]. To further confirm the cytotoxicity of berberine, we examined if the treatment can induce oxidative stress in SMMC-7721 and Bel-7402 cells. At a dose upper 100 μM, we found berberine treatment can initiate production and accumulation of ROS in cancer cells. This effect may be dose-independent, as berberine treatment at 100 and 200 μM led to comparable increase of intracellular ROS level. Pretreatment of 5 mM of *N*-Acetyl-l-cysteine (NAC) significantly attenuated the ROS level by BBR. The accumulation of ROS in cancer cells was coming up with shrinkage of cell shape, indicating cell death when cancer cells cannot overwhelm berberine-induced ROS production (Figure 2).

### 2.3. Migration Inhibition of HCC Cells by Low Dose Berberine (BBR)

**Figure 3:**
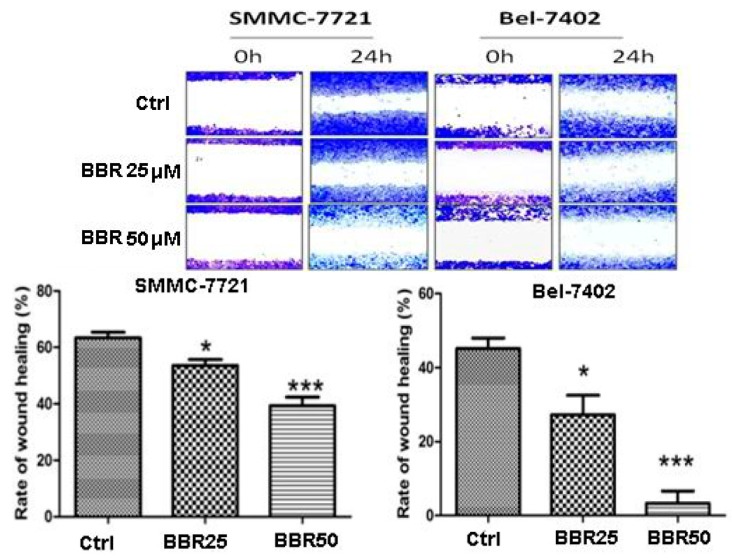
Decreased migration of BBR-treated HCC Cells by wound healing assay. SMMC-7721 and Bel-7402 cells were incubated in 12-well plates with wound healing assay inserts. BBR (25 and 50 μM) was added for 24 h. The cells were stained by violet crystal and the rate of wound healing was observed and calculated. * *p* < 0.05 (*vs.* the control group); *** *p* < 0.001(*vs.* the control group).

Our previous studies reported the non-toxic anti-tumor effect of berberine and *Coptidis Rhizoma*, whose major active component is berberine, on human cancer cells [33]. To elaborate whether low dose treatment of berberine can also affect SMMC-7721 and Bel-7402, we conducted wound healing assay to observe cell motility upon berberine treatment. Interestingly, instead of resulting in cell death, low dose treatment of berberine extremely reduced migration of human HCC cells. The movement of cancer cells towards center of the wounded gap was significantly restrained in the presence of berberine, indicating the migration of HCC cells was significantly inhibited (Figure 3).

### 2.4. Invasion Inhibition of HCC Cells by Low Concentration of BBR

**Figure 4:**
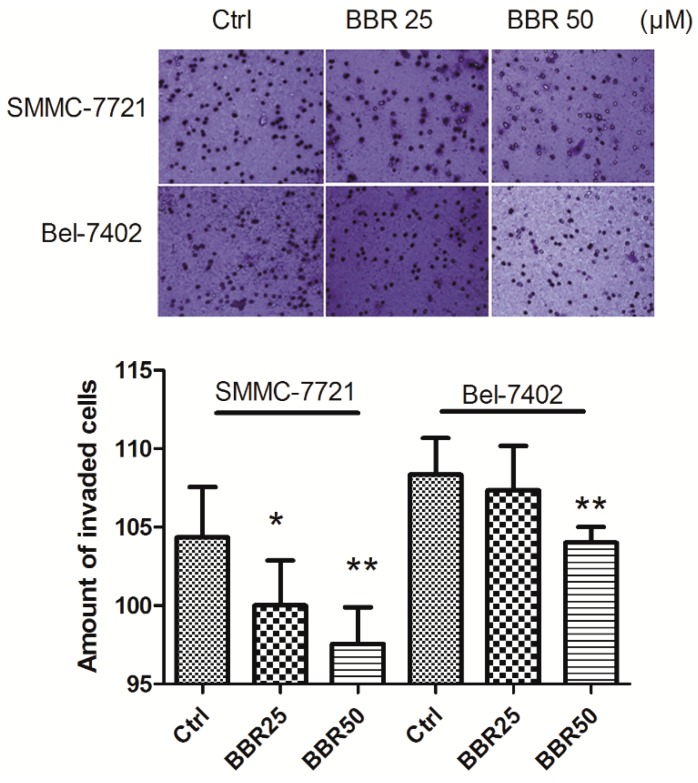
Decreased invasion of BBR-treated HCC cells by transwell test.Matrigel was coated on the transwell membranes. SMMC-7721 and Bel-7402 cells were diluted by 0.2% BSA-containing media in the upper of transwell. Media with BBR (25 and 50 μM) were added as attractants in the receiver wells. After 24 h, the invasive cells were calculated by violet crystal staining. * *p* < 0.05 (*vs.* the control group); ** *p* < 0.01(*vs.* the control group).

Motility of cancer cells is associated with its invasiveness through extracellular matrix. Metastasis of human HCC cells may request both high motility of HCC cell as well as the ability of tumor cell in degenerating extracellular matrix [34,35,36]. To further investigate the possible anti-metastasis effect of berberine in HCC cells, we conducted a transwell chamber assay to observe the effect of berberine on invasiveness of tumor cell through extracellular matrix. Matrigel was coated onto the upper inserts of the transwell, where cancer cell pretreated with the low dose of BBR (25 and 50 μM) for 24 h were seeded onto. Reduced invasion of berberine-treated HCC cells was observed after 24 h incubation, indicating that berberine treatment can significantly eliminate invasiveness of HCC cells (Figure 4).

### 2.5. Inhibition of Cyclooxygenase-2 (COX-2), NF-κB and Matrix Metalloproteinase (MMP)-9 in HCC Cells by Low Dose BBR

**Figure 5:**
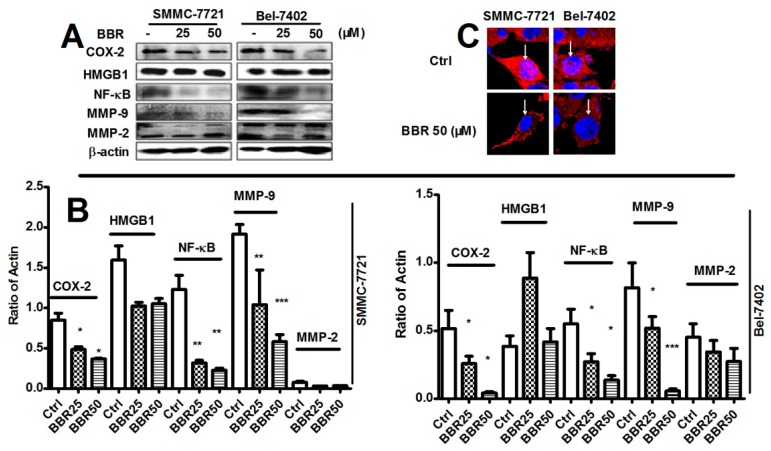
Inhibition of Cyclooxygenase-2 (COX-2), NF-κB and matrix metalloproteinase (MMP)-9 in BBR-treated HCC cells. SMMC-7721 and Bel-7402 cells were treated with BBR for 6 h. Proteins were collected. (**A**,**B**) expression of COX-2, HMGB1, NF-κB, MMP-9 and MMP-2 were analyzed by western blot. β-actin was used as internal standard. * *p* < 0.05 (*vs.* the control group); ** *p* < 0.01 (*vs.* the control group); *** *p* < 0.001(*vs.* the control group); (**C**) 50 μM of BBR-treated cells were incubated with NF-κB primary antibody, secondary antibody conjugated with Alexa Flour-568 (red dye). Nuclei were stained by DAPI (blue dye). NF-κB in both nuclei and cytoplasm was observed by confocal microscope. The results show that compared with the control group, the level of NF-κB (red) in nuclei (blue) was reduced (white arrow).

Berberine was reported as a potent inhibitor of *in vitro* and *in vivo* inflammation, which was recently identified as a key modulator in metastasis of HCC [37]. To identify the involvement of regulation on inflammation-associated pathways in the suppressive effect of berberine on HCC migration and invasion, we examined whether berberine can reduce expression of several inflammatory factors including Cyclooxygenase-2 (COX-2), high mobility group box 1 (HMGB1), NF-κB, matrix metalloproteinase (MMP)-9 and MMP-2. Interestingly, berberine can particularly reduce COX-2, NF-κB and MMP-9 expression, while no effects of berberine on HMGB1 and MMP-2 were found (Figure 5A,B). Furthermore, treatment of BBR resulted in export of NF-κB from nuclear region of the cells which inactivated NF-κB. This indicates that berberine is not a robust inhibitor of inflammation-related pathways but can target to some specific inflammatory molecules.

### 2.6. Inactivation of p38 and Erk1/2 MAPK in BBR-Treated HCC Cells

**Figure 6:**
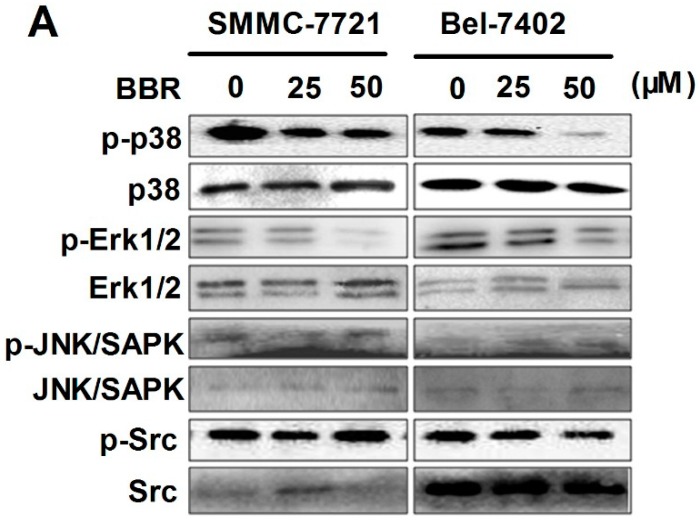

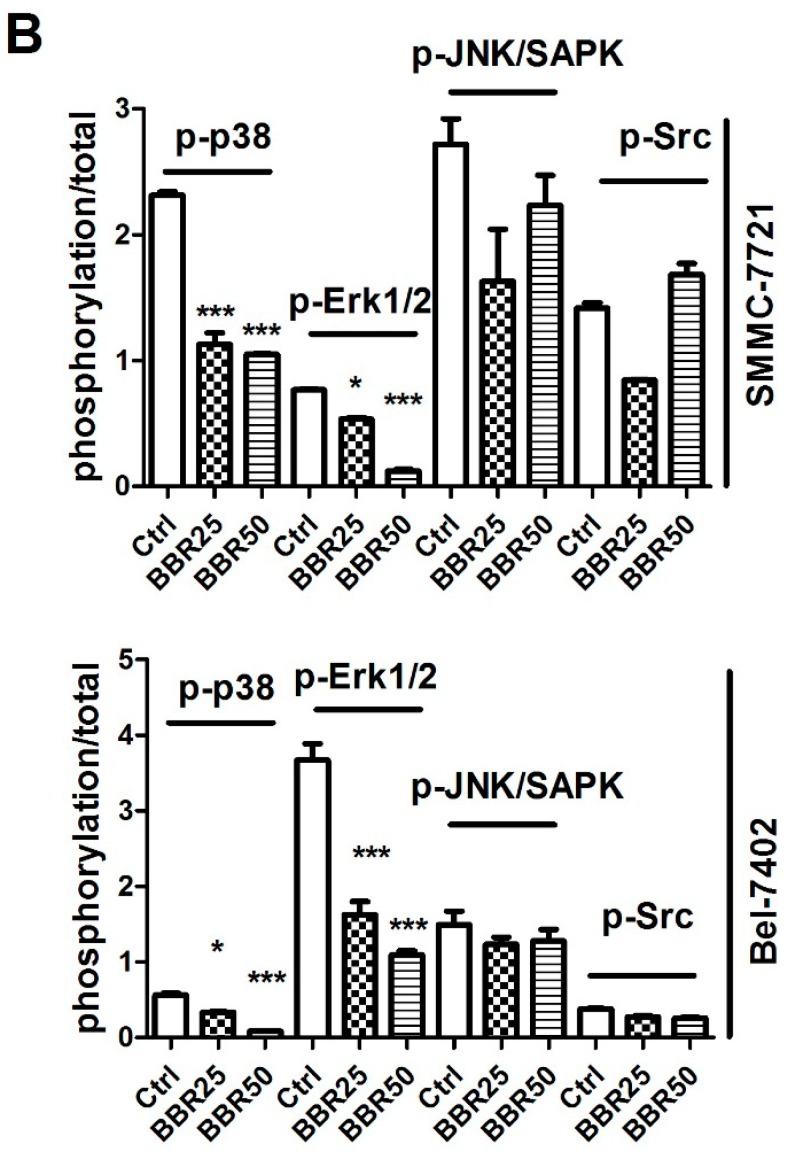
Inactivation of p38 and Erk1/2 in the low concentration of BBR-treated HCC cells by western blot. SMMC-7721 and Bel-7402 cells were treated with BBR for 6 h. Proteins were collected, determined and expression of p-p38, p-Erk1/2, p-JNK/SAPK and p-Src were analyzed by western blot. The expression of total proteins was used as internal standard. (**A**) Protein expression was observed by ChemiDoc™ XRS^+^ Molecular Imager; (**B**) Protein expression was calculated by ImageJ 1.38x software. * *p* < 0.05 (*vs.* the control group); *** *p* < 0.001(*vs.* the control group).

To identify the possible mechanism underlying berberine-suppressed expression of particular inflammatory factors in HCC cells, we screened several associated upstream signaling pathway including P38 MAPK, Erk1/2, Src and JNK/SAPK. It was found in both HCC cells that berberine significantly suppressed the phosphorylation of p38 MAPK and Erk1/2 pathways. However, berberine did not exhibit potent inhibition on Src and JNK/SAPK signaling. This data further proved our hypothesis that only particular inflammation-related pathways can be repressed by berberine treatment (Figure 6).

### 2.7. Berberine Inhibited Urokinase-Type Plasminogen Activator (uPA) Receptor via Inducing Plasminogen Activator Inhibitor-1 (PAI-1) Expression and Suppressing uPA in HCC Cells

**Figure 7:**
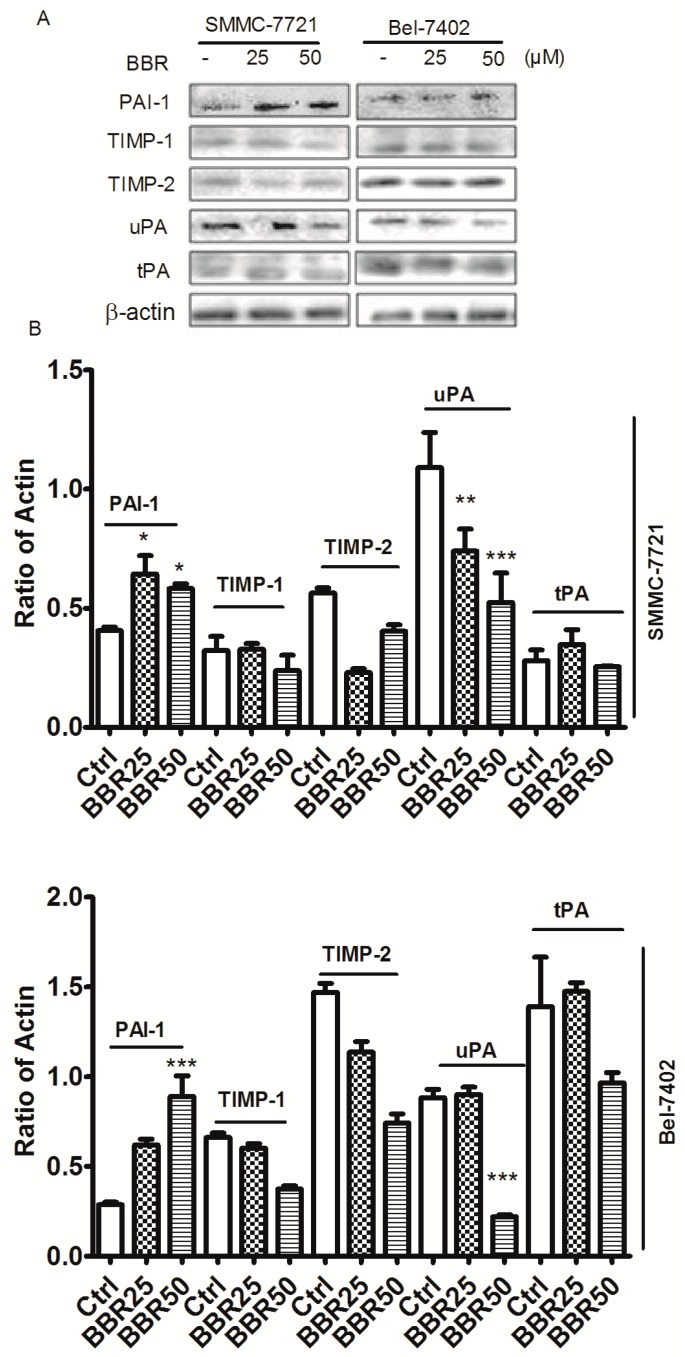
Induction of plasminogen activator inhibitor-1 (PAI-1) and inhibition of urokinase-type plasminogen activator (uPA) in BBR-treated HCC cells by western blot. SMMC-7721 and Bel-7402 cells were treated with BBR for 6 h. Proteins were collected, determined and expression of PAI-1, TIMP-1, TIMP-2, uPA and tissue-type plasminogen activator (tPA) were analyzed by western blot. β-actin was used as internal standard. (**A**) Protein expression was observed by ChemiDoc™ XRS^+^ Molecular Imager; (**B**) Protein expression was calculated by ImageJ 1.38x software. * *p* < 0.05 (*vs.* the control group); ** *p* < 0.01 (*vs.* the control group); *** *p* < 0.001 (*vs.* the control group).

In order to identify the common upstream regulatory molecules that involves in the inhibition of p38 MAPK and Erk1/2 in berberine-treated HCC cells, we examined expression of some possible regulator of the pathways including TIMP family, urokinase-type plasminogen activator (uPA) and PAI-1. Berberine treatment has minimal effect on the expression of TIMPs family such as TIMP-1 and TIMP-2. Particularly, expression of the uPA was dose-dependently reduced upon berberine treatment while tissue-type plasminogen activator (tPA) were not statistically changed. In addition, an endogenous antagonist PAI-1 was induced in berberine-treated cells (Figure 7).

## 3. Discussion

Previous studies showed that distant metastasis was the main factor that leads to failure in conquering HCC. During metastasis of cancer, tumor cell requires degeneration of extracellular matrix which facilitates its invasion towards nearby tissues. This is followed by penetrating blood vessels and migrate the distant organs [3,38]. In this case, the initiating and essential step of tumor cell invasion is to break through from the restriction of extracellular matrix by degrading local proteins. The most important executors for proteolysis are MMPs [34,35,36]. Furthermore, such process is controlled by some signaling pathways including PAI-1, tPA, uPA and TIMPs [39]. Our previous data showed that *Coptidis Rhizoma* extracts could inhibit the migration of human hepatocellular carcinoma cells MHCC97-L [17]. Furthermore, we found that BBR as low dose as to 50 μM inhibited migration of nasopharyngeal carcinoma HONE1 cells [18]. We hypothesized that it might be berberine, which is the major natural alkaloid in *Coptidis Rhizoma*, possesses the activity of inhibiting migration and invasion of HCC cells. However, it is still unclear how BBR affects migration and invasion in HCC. In this study, we found that BBR can dominantly induce HCC cell apoptosis at the concentration more than 100 μM and/or when the treatment lasted for more than 24 h. The intracellular ROS level was increased as well. As ROS generation mediates activation of an intrinsic apoptosis [26], this result offers evidence that sustained treatment of relative high dose of BBR induces ROS-associated intrinsic apoptosis in HCC cells.

In contrast, though low dose treatment of berberine appears to have minimal effect on cell viability of HCC cells, this treatment is enough to restrain migration and invasion of HCC cells, which was consistent with our previous observations [17,18] and other literature [40] on both berberine and *Coptidis Rhizoma*.

**Figure 8:**
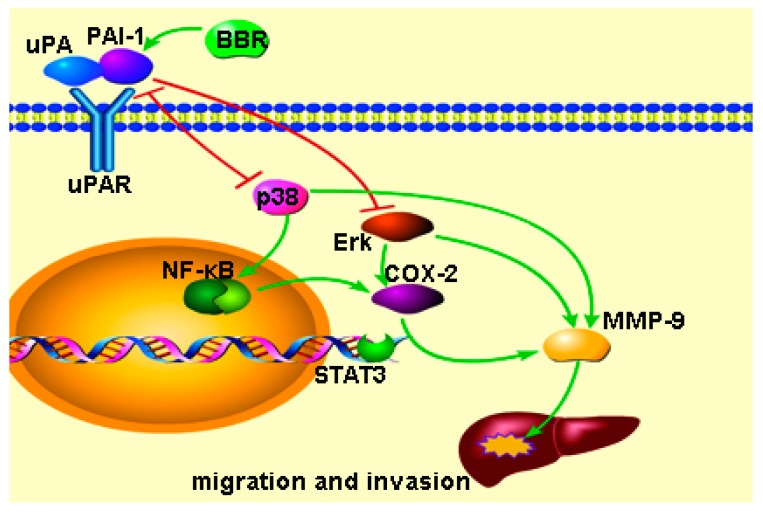
Schematic diagram of BBR-induced PAI-1 cell signaling pathway in HCC cells.

A body of evidence emphasizes that inflammation responses participate the different stages of cancer, including initiation, promotion, malignant conversion, invasion, and metastasis [20]. COX-2 can produce prostaglandins (PGs), which then contributes to cancer invasion and metastasis [37]. Of note, COX-2 is a one of the downstream genes of NF-κB, whose inactivation may consequently lead to inhibition of COX-2-induced cell invasion and migration [41,42]. On the other hand, MMPs were up-regulated by uPA and tPA and down-regulated by TIMPs and PAI-1. Both inhibiting uPA and tPA and activating PAI-1 and TIMPs can inhibit migration and invasion [39]. Our data in this study showed the expression of NF-κB, COX-2 and MMP-9 was suppressed upon berberine treatment, indicating that the anti-invasive effect of berberine may be associated with inflammation response of cancer cells upon treatment. Furthermore, berberine was found to inactivate p38 and Erk1/2 pathways, both of which belong to MAPK signaling pathway family. Studies showed that MAPK signaling pathways regulate a number of cancer cell behavior including migration and invasion [43]. MAPKs are downstream of several inflammation-associated receptors such as toll-like receptor 4 (TLR4) and uPA receptor (uPAR) [22,44,45]. In this study, the results indicated that BBR induced PAI-1 and inactivated p38 and Erk1/2 which may involve in inhibition of cancer cell migration and invasion. In particular, we found that berberine can induce expression of PAI-1, an important of antagonist of both TLR4 and uPAR. This means induction of PAI-1 may be involved in inactivation of p38 and Erk1/2 by BBR. In addition, HMGB1, another important inflammation factor, may involve cell migration and invasion by TRL4 [46]. HMGB1 may interact with PAI-1 which also participates TRL4 signaling pathway [44], thus we investigated if HMGB1 involved in BBR-induced PAI-1. However, the results showed there was no significantly change of HMGB1 in this study. Interestingly, decrease of uPA was observed in berberine-treated HCC cells. Evidence shows that uPAR overexpression can selectively induce the expression of active uPA by a feedback loop, and uPAR overexpression induces the activation of ERKs and p-38 MAPKs [47]. Taking together, these findings indicate that up-regulation of PAI-1 and down-regulation of uPA by berberine may inactivate uPAR and its downstream signaling, which in turn suppresses inflammation-associated migration and invasion of HCC cells. The regulatory scheme of berberine on PAI-1/uPA-associated pathway was shown in Figure 8.

However, the role of PAI-1 in cancer progression seems contradicted in different studies. The protein was found to inhibit cell migration/invasion [45,48], while in some other cases, PAI-1 was shown to promote oncogenesis and cell migration [44,49]. Nevertheless, silencing of PAI-1 suppresses cancer progression and liver metastasis [50]. The inconsistent conclusions from different laboratories might be due to a feedback loop between PAI-1 and MAPK. PAI-1 can block uPA and MMPs by inhibiting MAPK [45,48]. MAPKs also interact with PAI-1 [51] and consequently stimulate the increase both uPA and PAI-1 [52]. This feedback loop leads to data fluctuation when experimental conditional is not extremely the same. Additionally, PAI-1 is not merely produced by cancer cell itself. Many other types of cells such as platelet, macrophage and vascular cell can secrete PAI-1. Different source of PAI-1 may function different roles [53]. These potential factors should be paid attention to when PAI-1 is considered as a biomarker in clinics [52].

## 4. Materials and Methods

### 4.1. Experimental Agents

Berberine (1065210) and Primary antibodies uPA (SAB1105036), tPA (HPA003412), TIMP-2 (WH0007077M1) were purchased from Sigma-Aldrich Company (Sigma-Aldrich, St. Louis, MO, USA). COX-2 (#4842S), PAI-1 (#11907S), p-p38 (#4511), p38 (#8690), p-Erk1/2 (#4370), Erk1/2 (#4695), p-JNK/SAPK (#9255), p-Src (#12432), Src (#2108), HMGB1 (#6893S), NF-κB (#3033, #8242), TIMP-1 (#8946), MMP-9 (#3852S), MMP-2 (#4022S) and actin (#3700) were purchased from Cell Signaling Company (Cell Signaling, Danvers, MA, USA). JNK/SAPK (EPR18841-95) was purchased from Abcam (Cambridge, MA, USA). Secondary antibodies were purchased from Beyotime Institute of Biotechnology (Beyotime Inst. Biotech., Shanghai, China).

### 4.2. Cell Culture and Treatments

HCC cell lines Bel-7402 and SMMC-7721 were cultured by our lab. Cells were maintained in RPMI1640 medium (GIBCO, Carlsbad, CA, USA) supplemented with 10% FBS (Sijiqing Inc., Hangzhou, China) at 37 °C in a 5% CO_2_ humidified incubator. Cells in the logarithmic growth phase were used in all experiments.

### 4.3. Experimental Design

Human hepatocellular carcinoma cells Bel-7402 and SMMC-7721 were divided to the control (Ctrl), BBR (25, 50, 100 and 200 μM) groups, respectively. Except the control group, HCC cells were treated with the different concentrations of BBR.

### 4.4. Cell Viability Assay

Cells were plated in 96-well plates at a density of 5000 cells/well and cultured for 24 h. After culturing cells with/without various concentration of BBR (0, 6.25, 12.5, 25, 50, 100 and 200 μM), the solution of 3-(4,5-dimethylthiazol-2-yl)-2,5-diphenyltetrazoli-um bromide (MTT) was added as a quantitative colorimetric assay dye. DMSO was then added to resolve the crystal following the remove of the media. The optical density (OD) value was measured at 540 nm with a microplate reader (TECAN, Mannedorf, Switzerland).

### 4.5. Intracellular Level of ROS Detection

To investigate the cause of high concentration of BBR inducing HCC cell apoptosis, intracellular ROS generation was determined by fluorescent microscopy. HCC cells were cultured with/without BBR (100 and 200 μM) in 6-well plates for 6 h. As a negative control group, 5 mM of NAC (Beyotime Inst. Biotech.) was added into HCC cells to attenuate the effects by 200 μM BBR. 2,7-dichlorodihydrofluorescein diacetate (DCFDA, ROS dye) and Hoechst 33258 (nucleic dye) (Sigma-Aldrich) were added into cells. The intracellular ROS level was observed by detecting the fluorescent density. The intracellular ROS level was observed by fluorescent microscopy (Nikon TE2000, Tokyo, Japan).

### 4.6. Wound Healing Assay

HCC cells were suspended at 0.5 × 10^6^ cells/mL and 500 μL of the cell suspension to each well was cultured in CytoSelect™ Wound Healing Assay Kit (CBA-120, Cell Biolabs, Inc., San Diego, CA, USA). For the intervention by BBR (25 and 50 μM), the inserts were located for 24 h. The cells were stained by 1% violet crystal and the cell migration rate was observed and calculated according to the protocol.

### 4.7. Transwell Assay

Briefly, the transwell inserts (NO.3422, Corning, Troy, MI, USA) were coated with BD Matrigel™ (BD, Bedford, MA, USA) under sterile conditions in 24-well transwell plate and incubated in 5% CO_2_ incubator at 37 °C overnight and re-coated with BD Matrigel™ for 30 min before use. Each well was blocked with RPMI-1640 medium (containing 10 mg/mL BSA 50 μL) for 30 min. 5 × 10^5^ cells/mL of HCC cells 100 μL in 0.2% BSA-containing medium were cultured in the upper of the tranwells while 600 μL medium (with 10% FCS) was added in the receiver wells. HCC cells were divided into the control (Ctrl), BBR 25 μM (BBR25), BBR 50 μM (BBR50) groups and incubated for 24 h. Violet crystal was used for staining and calculating the invasive cell number. The average number was calculated from the five equal field under light microscopy.

### 4.8. Western Blot

Protein samples were quantified and stored at −80 °C until use. The electrophoresis was conducted on a SDS-PAGE gel at 120 V using Powerpac Basic electrometer (Bio-Rad, Hercules, CA, USA) following the Bio-Rad Laboratories handbook. Proteins from the gel was transferred into a PVDF membrane paper at 70 V for 2 h by using a Fast-Transfer Blot System (Bio-Rad). The membrane was blocked by 5% BSA and incubated with the primary antibodies on a rocker overnight, followed by incubating with the secondary antibodies on a rocker for 1 h. After rinsing four times (10 min × 1 and 5 min × 3), the membrane was developed by Millipore ECL chemiluminescence kit (Millipotre Corporation, Billerica, MA, USA) and exposure in ChemiDoc™ XRS^+^ Molecular Imager (Bio-Rad).

### 4.9. Confocal Microscope

HCC cells were treated with 50 μM of BBR for 6h and were fixed with 4% paraformaldehyde and penetrated with 0.3% Triton-X100. Followed by incubated with block buffer (PBS containing 10% normal goat serum), cells were incubated with NF-κB antibody (#8242, 1:200, Cell signaling technology, Danvers, MA, USA) overnight at 4 °C. Cells were then washed with PBS, incubated with secondary antibody conjugated with Alexa Flour-568 (red, 1:500, Life Technologies, Waltham, MA, USA). Nuclei was stained with DAPI (blue). Image was captured by using LSM780 confocal microscope (Carl Zeiss, Goettingen, Germany).

### 4.10. Statistical Analysis

All the experiments were performed in triplicate. The data were expressed as mean ± standard deviation and statistical significance was determined by One-way analysis of variance (ANOVA) followed by Turkey’s test by using the software SPSS 17.0 (SPSS Inc., Chicago, IL, USA). *p* < 0.05 was considered as statistical significance.

## 5. Conclusions

High dose of BBR can induce cell apoptosis by ROS generation while low dose of BBR can inhibit migration and invasion of HCC cells. This anti-invasion and anti-migration effect of berberine may be associated with suppression of inflammation response of HCC cells, as evidenced by reduced expression of COX-2 and MMP-9. Inhibition of p38 and Erk1/2 MAPK signaling pathways was observed in berberine-treated HCC cells. The upstream molecules of MAPKs family, uPAR was inactivated by up-regulation of PAI-1 and down-regulation of uPA. Our findings primarily unveiled for the first time that suppression of uPA receptor-associated inflammation response is involved in the inhibitory effect of berberine in cancer cell migration and invasion.
